# Three-dimensional analysis of the anterior loop of the inferior alveolar nerve in relation to the growth pattern of the mandibular functional subunit

**DOI:** 10.1186/s40902-018-0170-4

**Published:** 2018-11-05

**Authors:** Seungkyu Yoon, Jae-Young Kim, Cheol-Hee Jeong, Jengbin Park, Jong-Ki Huh, Kwang-Ho Park

**Affiliations:** 1 Department of Oral and Maxillofacial Surgery, Gangnam Severance Hospital, Yonsei University College of Dentistry, 211 Eonju-ro, Gangnam-gu, Seoul, 06273 South Korea; 2 Department of Dentistry, Armed Forces Guri Hospital, Guri-si, Korea

**Keywords:** Mandibular nerve, Genioplasty, Computed tomography, Chin, Chin repositioning

## Abstract

**Background:**

The purpose of the present study was to investigate the differences in the position and shape of the anterior loop of the inferior alveolar nerve (ALIAN) in relation to the growth pattern of the mandibular functional subunit.

**Methods:**

The study was conducted on 56 patients among those who had undergone orthognathic surgery at the Gangnam Severance Hospital between January 2010 and December 2015. Preoperative computerized tomography (CT) images were analyzed using the Simplant OMS software (ver.14.0 Materialise Medical, Leuven, Belgium). The anterior and inferior lengths of ALIAN (dAnt and dInf) and each length of the mandibular functional subunits were measured. The relationship between dAnt, dInf, and the growth pattern of the mandibular subunits was analyzed.

**Results:**

The length of the anterior portion of ALIAN (dAnt) reached 3.34 ± 1.59 mm in prognathism and 1.00 ± 0.97 mm in retrognathism. The length of the inferior portion of ALIAN (dInf) reached 6.81 ± 1.33 mm in prognathism and 5.56 ± 1.34 mm in retrognathism. The analysis of Pearson’s correlation coefficiency on all samples showed that the lengths of functional subunits were positively correlated with the loop depth. The length of the symphysis area in prognathic patients was positively correlated with the anterior loop depth (*p* = 0.005).

**Conclusions:**

Both the anterior and inferior length of ALIAN are longer in prognathic patients. Especially, it seems to be associated with the growth of the symphysis area.

## Background

Iatrogenic damage to the inferior alveolar nerve (IAN) can cause temporary or permanent discomfort in the lower labium and labial gingival, and therefore, accurate preoperative identification of its location is imperative. Although the location of the mental foramen can be predicted through clinical and radiological means, the anterior loop area of the mandibular canal can often be difficult to identify. Because of this, unintended damage to the inferior alveolar nerve can occur while performing implantation, open reduction, and genioplasty on the mandibular premolar region. Precedent studies have reported pre- and postoperative inferior alveolar nerve damage rates of 8.5~24% during implant placement [1–4] and 17~38% during genioplasty [5–8].

It has been reported that the location of the mental foramen may vary according to the pattern of growth and asymmetry of mandibular growth and various studies have reported the length of the anterior loop of the mandibular canal itself to reach 0.11~10 mm [9–11].

The present study aimed to investigate the differences in the position and shape of the anterior loop of inferior alveolar nerve (ALIAN) in relation to the growth pattern of the mandibular functional subunit.

## Methods

### Subjects

Among 417 patients who underwent orthognathic surgery between January 2010 and December 2015 at the Department of Oral and Maxillofacial Surgery, Gangnam Severance Hospital, those who had taken preoperative CT images were selected as the study candidates. Among them, those who underwent concomitant TMJ open surgery, such as total joint replacement or arthroplasty, were excluded, and patients in whom canal location could not be identified on CT due to low bone density were also excluded. Ultimately, a total of 56 patients (28 with retrognathism and 28 with prognathism) were included.

### Reference points, line, and planes (Table 1)

Nasion (Na), porion (Po), orbitale (Or), pogonion (Pog), mental foramen (MF), gonion (Go), and sigmoid notch (SN) were set as the reference points (Table 1). FH line was drawn connecting the center of the porion (PoC) and orbitale (OrC), which were automatically determined. McNamara plane (N-perpedicular plane, pMcN) was defined as the plane perpendicular to the FH line and bypassing the Na [12]. To evaluate the anterior and inferior distance of the anterior loop of IAN, occlusal plane (pOcc) and planes passing through MF (pMF_H and pMF_V) were also determined. They were summarized in Table 1.

**Table 1:** The reference point, line, and the plane

Landmarks	Abbreviation	Description
Point		
Nasion	Na	The middle point of the junction of the frontal and the two nasal bones
Porion	Po	The highest point of the skeletal external auditory meatus (PoR, right side; PoL, left side; PoC, center of the PoR and PoL, automatically determined)
Orbitale	Or	The lowest point in the lower margin of the bony orbit (OrR, right side; OrL, left side; OrC, center of the OrR and OrL, automatically determined)
Pogonion	Pog	The most anterior point of the mandible in the midline
Mental foramen	MF	The center of mental foramen (MFR, right side; MFL, left side)
Gonion	Go	The most inferior, posterior, and lateral point of the angle of the mandible (GoR, right side; GoL, left side)
Sigmoid notch	SN	The deepest point between the condylar and coronoid process of the mandible (SNR, right side; SNL, left side)
Line		
FH line		A line projecting the PoC and OrC
Plane		
McNamara plane	pMcN	A plane through the nasion and perpendicular to the FH line
Occlusal plane	pOcc	A plane through the bilateral mandibular first molar mesiobuccal cusp and the middle of the lower incisal edge
MF_H plane	pMF_H	A plane through the MF point and parallel to the pOcc
MF_V plane	pMF_V	A plane through the bilateral MF and perpendicular to the pOcc

### Measurement of mandibular functional unit (Table 2)

The diagnosis of dentofacial deformity was made according to three-dimensional McNamara analysis using Simplant OMS (ver.14.0, Materialise Medical, Leuven, Belgium) [12]. Mandibular prognathism was defined when Pog was located more than 2 mm anteriorly to the pMcN. Conversely, if Pog is located more than 2 mm posteriorly to the pMcN, it was diagnosed as mandibular retroganthism [13].

**Fig. 1:**
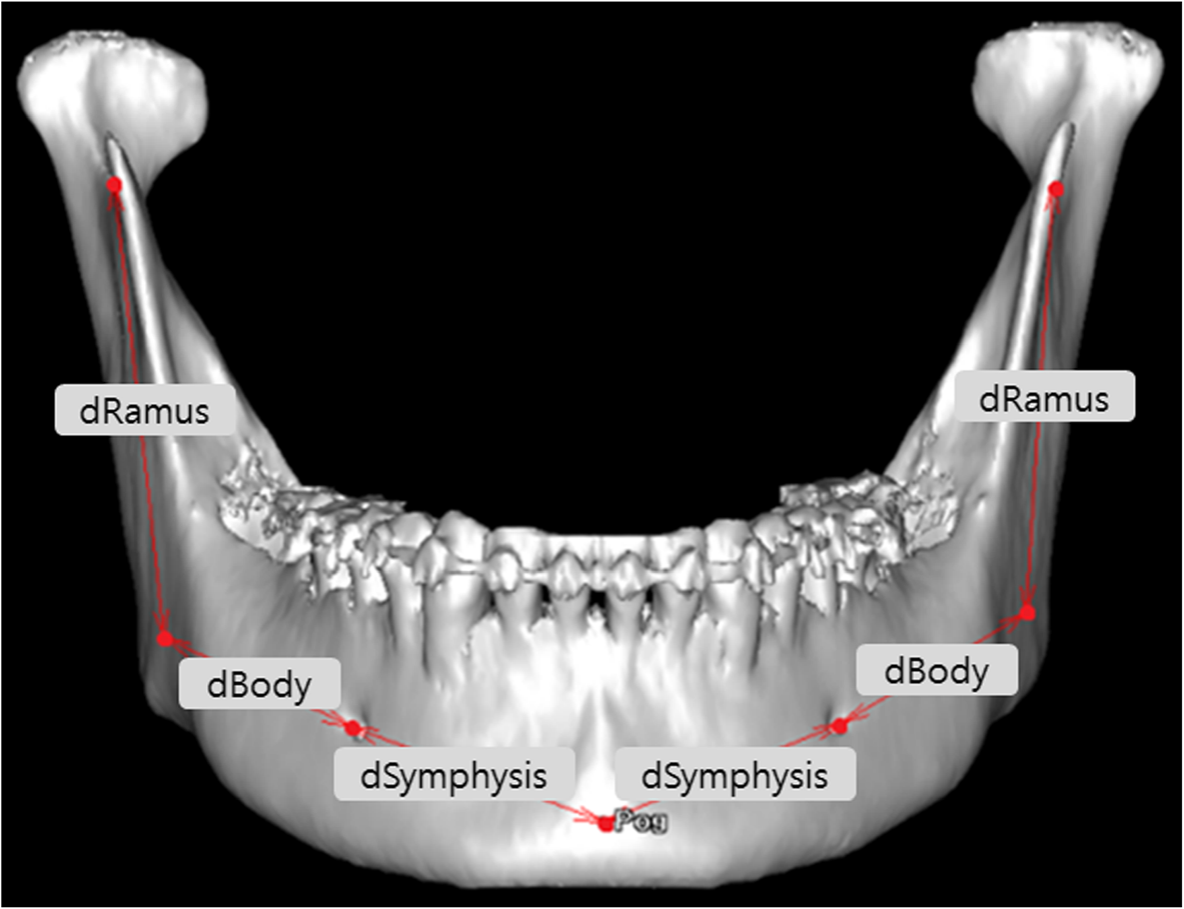
Functional subunit of the mandible. dRamus, distance from the sigmoid notch to the gonion; dBody, distance from the gonion to the MF point; dSymphysis, distance from the MF point to the pogonion

The most posterior, inferior point of the mandibular body was defined as gonion (Go), and the distance from the most inferior point of the sigmoid notch (SN) to Go, from Go to MF, and from MF to Pog was defined as dRamus, dBody, and dSymphysis, respectively (Fig. 1, Table 2).

**Table 2:** Distance measurements of loop of the inferior alveolar nerve and the mandibular functional units

Measurement	Description
dAnt	Distance from the most anterior point of the inferior alveolar nerve to pMF_V (If there is no anterior curvature, dAnt is defined as “0”)
dInf	Distance from the most inferior point of the inferior alveolar nerve to pMF_H
dPog	Distance from the Pog to the pMcN
dRamus	Distance from the SN to the Go
dBody	Distance from the Go to the MF
dSymphysis	Distance from the MF point to the Pog

### Reconstruction and measurements of the anterior loop of IAN

For the inferior alveolar nerve reconstruction, a single researcher marked the midpoint of the inferior alveolar nerve canal on each plane by visual inspection, and reconstruction was performed by using the create nerve function in the software.

**Fig. 2:**
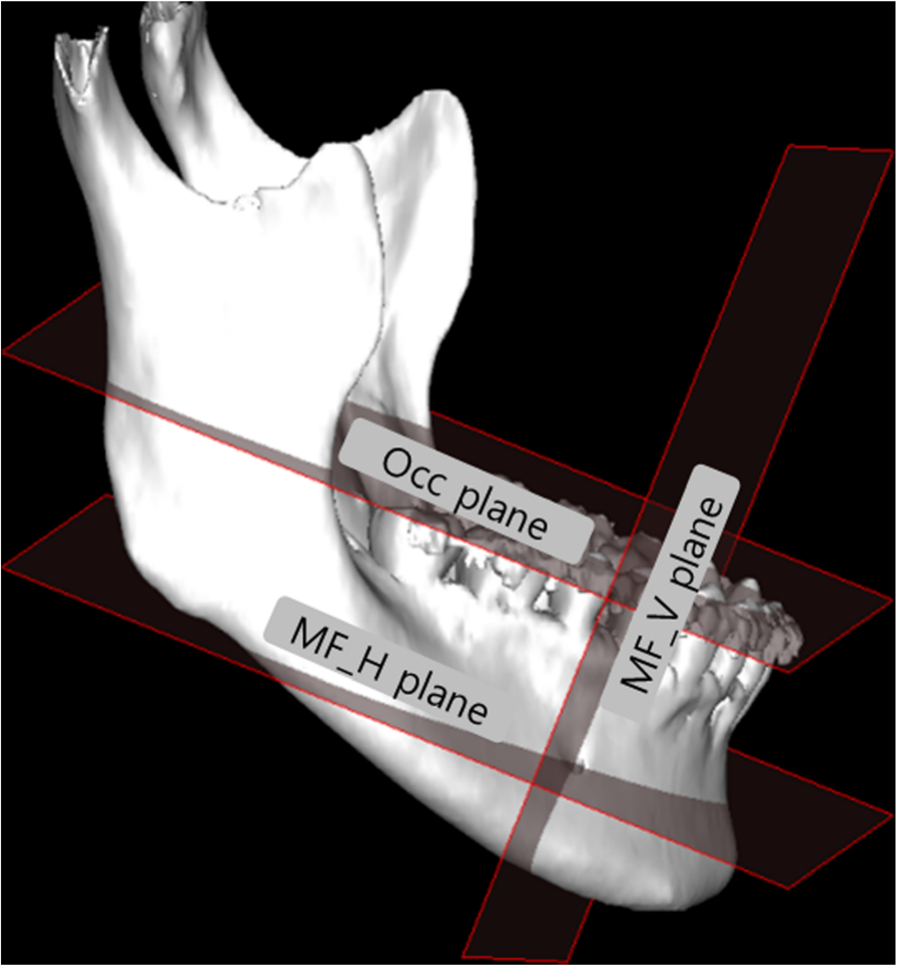
Occ, MF_V, and MF_H plane. Occ plane, a plane through the bilateral mandibular first molar mesiobuccal cusp and the center of the lower incisal edge; MF_V plane, a plane through the MF point and parallel to the Occ plane; MF_H plane, a plane through the bilateral MF point and perpendicular to the Occ plane

A plane bypasses the mesiobuccal cusps of bilateral mandibular first molars, and the midpoint of the mandibular incisal edge was defined as the occlusal plane (pOcc). A plane perpendicular to the occlusal plane and bypassing MF points was defined as pMF_V. A plane parallel to Occ plane and bypassing MF points was defined as pMF_H (Fig. 2). The planes of right and left sides were determined separately.

**Fig. 3:**
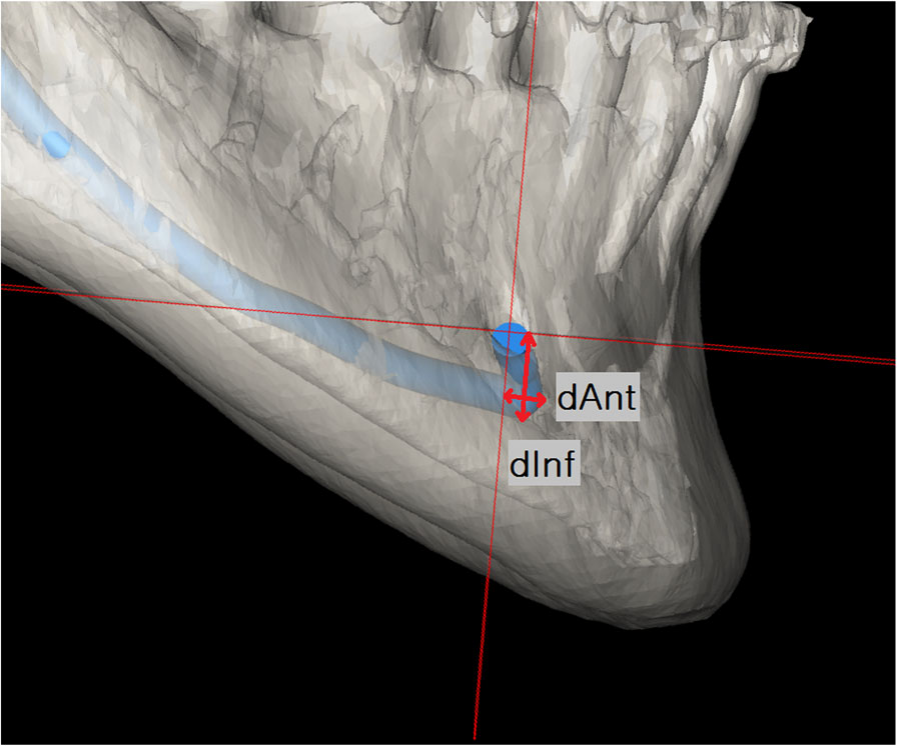
Measurement of dAnt and dInf. dAnt, distance from the most anterior point of the inferior alveolar nerve from MF_V. If there is no anterior curvature, dAnt is defined as “0”; dInf, distance from the most inferior point of the inferior alveolar nerve from MF_H

With respect to the loop, distance from the most anterior point of reconstructed IAN to pMF_V (dAnt) and distance from the most inferior point of reconstructed IAN to pMF_H (dInf) were measured on both the left and right side. In cases where the loop was not deep enough so that it was positioned anteriorly to MF-V plane, dAnt was assumed to be 0 (Fig. 3).

### Statistical analyses

All statistical analyses were performed using SPSS Statistics v23.0 (IBM Corp). Student’s *t* tests were performed on the dAnt and dInf. Pearson’s correlations of the lengths of each mandible subunits were analyzed in relation to the dAnt and dInf.

## Results

The subjects of the study consisted of a total of 56 patients; 22 males and 34 females, with a mean age of 26.14 ± 5.79 years. Among them, there were 28 mandibular prognathism (14 males and 14 females, mean age of 23.11 ± 2.42 years) and 28 mandibular retrognathism (8 males and 20 females, mean age of 29.18 ± 6.58 years). The distance from the pMcN to Pog was 10.20 ± 4.73 mm in prognathism and − 19.12 ± 8.13 mm in retrognathism.

In prognathic patients, dAnt was 3.34 ± 1.59 mm and dInf was 6.81 ± 1.33 mm, with maximum value for each being 7.12 mm and 10.38 mm, respectively. In retrognathic patients, dAnt was 1.00 ± 0.97 mm and dInf was 5.56 ± 1.34 mm, with maximum value for each being 2.89 mm and 8.68 mm, respectively (Table 3).

**Table 3:** Comparison of average distance of dAnt and dInf between prognathism and retrognathism (mm)

	Mean ± SD	Minimum	Maximum	*p* value
dAnt				
Prognathism	3.34 ± 1.59	0.51	7.12	< 0.001
Retrognathism	1.00 ± 0.97	0	2.89	
dInf				
Prognathism	6.81 ± 1.33	3.89	10.38	< 0.001
Retrognathism	5.56 ± 1.34	2.87	8.68	

*dPog* distance from the pogonion to the Mcnamara plane, *dAnt* distance from the most anterior point of the inferior alveolar nerve from the MF_V. If there is no anterior curvature, dAnt is defined as “0”, *dInf* distance from the most inferior point of inferior alveolar nerve from MF_H

The length of each mandibular functional subunit has a positive correlation with dAnt and dInf when evaluating both retrognathic and prognathic patients (Table 4). In retrognathism, there was no significant difference between the length of mandibular subunit and dAnt and dInf (Table 5) except dInf and dRamus, whereas prognathism patients showed a statistically significant correlation coefficient value 0.368 between symphysis and dAnt and 0.397 between symphysis and dInf (Table 6).

**Table 4:** Pearson’s correlation coefficiency in all patients

	dRamus	dBody	dSymphysis	dAnt	dInf
dAnt					
Co	.274	.510	.505		.502
*p*	.003**	.000*	.000*		.000*
dInf					
Co	.401	.336	.347	.502	
*p*	.000*	.000*	.000*	.000*	

*dAnt* distance from the most anterior point of the inferior alveolar nerve from MF_V. If there is no anterior curvature, dAnt is defined as “0,” *dInf* distance from the most inferior point of the inferior alveolar nerve from MF_H, *dRamus* distance from the sigmoid notch to the gonion, *dBody* distance from the gonion to the MF point, *dSymphysis* distance from the MF point to the pogonion, *Co* Pearson’s correlation coefficiency

*p < 0.001

**p < 0.05

**Table 5:** Pearson’s correlation coefficiency in retroganthic patients

	dRamus	dBody	dSymphysis	dAnt	dInf
dAnt					
Co	− .078	.219	.047		.058
*p*	.566	.105	.733		.673
dInf					
Co	.355	.001	− .065	.058	
*p*	.007*	.995	.636	.673	

*dAnt* distance from the most anterior point of the inferior alveolar nerve from MF_V. If there is no anterior curvature, dAnt is defined as “0”, *dInf* distance from the most inferior point of the inferior alveolar nerve from MF_H, *dRamus* distance from the sigmoid notch to the gonion, *dBody* distance from the gonion to the MF point, *dSymphysis* distance from the MF point to the pogonion, *Co* Pearson’s correlation coefficiency

*p < 0.05

**Table 6:** Pearson’s correlation coefficiency in prognathic patients

	dRamus	dBody	dSymphysis	dAnt	dInf
dAnt					
Co	.133	.134	.368		.502
*p*	.330	.325	.005*		.000*
dInf					
Co	.241	.200	.397	.502	
*p*	.074	.140	.002*	.000*	

*dAnt* distance from the most anterior point of the inferior alveolar nerve from MF_V. If there is no anterior curvature, dAnt is defined as “0”, *dInf* distance from the most inferior point of the inferior alveolar nerve from MF_H, *dRamus* distance from the sigmoid notch to the gonion, *dBody* distance from the gonion to the MF point, *dSymphysis* distance from the MF point to the pogonion, *Co* Pearson’s correlation coefficiency

*p < 0.05

## Discussion

The location of the inferior alveolar nerve acts as a limiting factor when various surgical approaches are attempted on the mandible. Because the nerve changes direction right before it comes out of the mental foramen and forms an anterior loop, it is necessary to create a safety margin that is more anterior and inferior to the location of the mental foramen when attempting a surgical approach on the premolar region or anterior mandible. However, the location of the mental foramen can be predicted by clinical and radiological means, whereas the identification of the anterior loop of the mandibular canal is often difficult. If iatrogenic damage to the inferior alveolar nerve occurs from the inability to identify the location of the anterior loop, it can lead to temporary or permanent discomfort in the lower labium and labial gingival. Various studies have reported pre- and postoperative inferior alveolar nerve damage rates of 8.5~24% during implant placement [1–4] and 17~38% during genioplasty [5–8].

The association between the location of the mental foramen and the pattern of the mandibular growth was proven by the functional matrix theory. The direction of the mental foramen is pointed coronally upon birth but gradually moves laterally as the mandible grows [14]. The present study assumed that as the direction of the mental foramen changed, the shape and length of inferior alveolar nerve anterior loop would also change accordingly, based on which comparative analysis was performed on the pattern of mandibular growth and the shape of inferior alveolar nerve anterior loop.

The inferior alveolar nerve anterior loop was located more anteriorly and inferiorly in prognathic patients (dAnt = 3.34 ± 1.59, dInf = 1.00 ± 0.97) than in retrognathic patient (dAnt = 6.81 ± 1.33, dInf = 5.56 ± 1.34). The absence of anterior loop was found in 22 canals (dInf = 0), all of which were identified in the retrognathic mandible.

At this study, there were prognathic cases which the loop was located inferiorly at a depth of ≥ 10 mm, which suggested that the safety margin of 3 mm generally used in genioplasty [15] may not be enough.

Correlation analyses performed separately for prognathic and retrognathic patients (Tables 5 and 6) showed that the anterior and inferior depth of the anterior loop was positively correlated with the length of symphysis in prognathic patients, whereas the length of each of the mandibular subunits did not show statistically significant correlations with each other in retrognathic patients. These results suggested that the growth in the symphysis area can affect the formation of the anterior loop during the mandibular growth process.

Correlation analysis performed on both prognathic and retrognathic mandibles together (Table 4) showed that the length of each mandibular subunit was highly correlated with the depth of the anterior loop. Therefore, it was demonstrated that, in general, as the length of each mandibular area becomes longer, the length of the anterior loop can also be longer as well.

## Conclusion

According to our study, especially, the anterior loop of inferior alveolar nerve was located more anteriorly and inferiorly in prognathic patients. It seems to be associated with the growth of the symphysis area. Therefore, surgeons should take care to avoid nerve damage at genioplasty or implant placement in the symphysis area in prognathism patients.

## Abbreviations

ALIAN: Anterior loop of the inferior alveolar nerve
dAnt: Distance from the most anterior point of the inferior alveolar nerve from MF_V
dBody: Distance from the gonion to the MF point
dInf: Distance from the most inferior point of the inferior alveolar nerve from MF_H
dRamus: Distance from the sigmoid notch to gonion
dSymphysis: Distance from the MF point to the pogonion
FH line: Frankfort horizontal line
Go: Gonion
GoL: Left side of the gonion
GoR: Right side of the gonion
MF: Mental foramen
Na: Nasion
Or: Orbitale
OrC: Center of OrR and OrL
OrL: Left side of the orbitale
OrR: Right side of the orbitale
pMcN: Macnamara plane
pMF_H: A plane through the MF point and parallel to the the pOcc
pMF_V: A plane through the bilateral MF and perpendicular to the pOcc
Po: Porion
PoC: Center of PoR and PoL
pOcc: Occlusal plane
Pog: Pogonion
PoL: Left side of the porion
PoR: Right side of the porion
SN: Sigmoid notch
SNL: Left side of the sigmoid notch
SNR: Right side of the sigmoid notch
